# Spontaneous emergence of fast attractor dynamics in a model of developing primary visual cortex

**DOI:** 10.1038/ncomms13208

**Published:** 2016-10-31

**Authors:** Thomas Miconi, Jeffrey L. McKinstry, Gerald M. Edelman

**Affiliations:** 1 The Neurosciences Institute, 800 Silverado Street, Suite 302, La Jolla, California 92037-4234, USA

## Abstract

Recent evidence suggests that neurons in primary sensory cortex arrange into competitive groups, representing stimuli by their joint activity rather than as independent feature analysers. A possible explanation for these results is that sensory cortex implements attractor dynamics, although this proposal remains controversial. Here we report that fast attractor dynamics emerge naturally in a computational model of a patch of primary visual cortex endowed with realistic plasticity (at both feedforward and lateral synapses) and mutual inhibition. When exposed to natural images (but not random pixels), the model spontaneously arranges into competitive groups of reciprocally connected, similarly tuned neurons, while developing realistic, orientation-selective receptive fields. Importantly, the same groups are observed in both stimulus-evoked and spontaneous (stimulus-absent) activity. The resulting network is inhibition-stabilized and exhibits fast, non-persistent attractor dynamics. Our results suggest that realistic plasticity, mutual inhibition and natural stimuli are jointly necessary and sufficient to generate attractor dynamics in primary sensory cortex.

## Abstract (web summary)

Sensory cortices represent stimuli through joint activity of competing neuronal assemblies. Here the authors show that a model of visual cortex with plastic feedforward and recurrent synapses, exposed to natural images, spontaneously develops attractor dynamics between groups of similarly tuned neurons.

## Graphical abstract

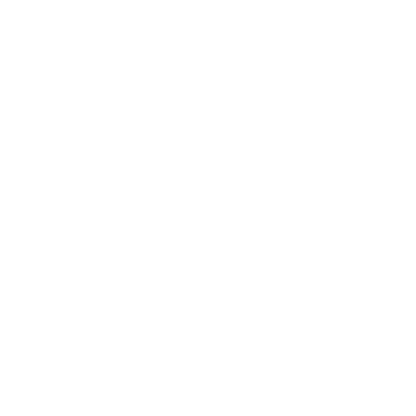

Sensory neurons are often studied for their properties as individual feature analysers^1^,^2^,^3^,^4^. However, recent evidence suggests that sensory neurons form coherent groups, which represent stimuli by their collective activity besides their individual responses. Bathellier *et al*.^5^ showed that local cortical microcircuits in mouse primary auditory cortex (∼70 cells) were constrained into a small number of possible response patterns (typically one or two, sometimes three), each associated with a subset of stimuli. This small number of response patterns is surprising, since with even a simple binary on-or-off readout, a population of *N* cells could in principle produce 2^*N*^ different response patterns. Importantly, the patterns were competitive and all-or-none: mixed stimuli evoked only one of the possible response patterns, rather than blended responses, with sharp transitions as the mixture of stimuli varied. These results expand and strengthen previous findings by Luczak *et al*.^6^ that the joint firing rates of local neurons only occupy a subspace of all possible patterns, both in stimulus-evoked responses and in spontaneous activity. Similarly, in mouse visual cortex, Miller *et al*.^7^ showed that neurons tended to respond as ‘cortical ensembles' of jointly firing neurons, with a simultaneity that could not be explained solely by the response properties of individual neurons. Furthermore, the same ensembles observed in evoked responses were also observed during spontaneous activity, emphasizing their intrinsic nature; this confirms previous findings that spontaneous, stimulus-absent cortical activity resembles stimulus-evoked responses^8^,^9^,^10^.

There are several possible explanations for this group behaviour among sensory cortical neurons. It might be inherited from upstream sources (sensory or thalamic); or it might reflect common distant inputs for each group of neurons. An alternative possibility, however, is that the group behaviour among sensory neurons actually emerges within cortex from attractor dynamics, in which local connectivity automatically drives population activity towards one of a few stereotypical patterns.

Attractor dynamics in recurrent neural networks have been thoroughly studied in the context of auto-associative memory, in which stimulus-selective assemblies show persistent activity after stimulus offset^11^,^12^,^13^,^14^,^15^, possibly with slow switching dynamics between the groups^16^,^17^. Such persistent-activity networks suggest a natural formation mechanism, namely Hebbian plasticity forming symmetric excitatory connections between similarly responding cells. Symmetric connections ensure that network activity descends the gradient of a well-defined potential (or Hamiltonian^11^), with the minima of this potential acting as attractors. Interestingly, recent studies of anatomical and functional connectivity in sensory cortical microcircuits are highly consistent with such attractor network configurations. Connections between nearby excitatory cells tend to be bidirectional, sparse and cliquish^18^. Symmetric connectivity also extends to inhibitory cells, whose connectivity with nearby principal cells is largely all-to-all and nonspecific^19^,^20^. Furthermore, lateral connections between nearby excitatory cells preferentially link cells with similar tuning^21^,^22^ and the lateral, intra-cortical input to principal cells has tuning similar to their feedforward, geniculate input^23^,^24^,^25^, which is precisely the expected pattern in an attractor network organized into a number of reciprocally connected, jointly active neuronal groups.

However, the concept of attractor dynamics in primary sensory cortex (as opposed to memory or cognitive areas) remains controversial. First, requirements for primary sensory cortices are very different from those for memory networks. The hallmark of sensory cortex is precisely to show much reduced activity in the absence of stimuli, in opposition to the persistent stimulus-absent activity that characterizes memory networks. In particular, Goldberg *et al*.^26^ point out that attractor-generating recurrent excitatory connectivity would also cause a slowing of network dynamics (that is, persistence), to an extent that is difficult to reconcile with observations. By contrast, Murphy and Miller^27^ argue that, under strong inhibition by a separate population of inhibitory neurons, appropriate network connectivity can generate attractor dynamics with fast fluctuations, transitions and decay, in accordance with observations; however, their simulations use hand-tuned connectivity, leaving open the question of how the required connectivity might arise.

Furthermore, primary sensory cortex neurons also possess highly selective receptive fields, which dynamically adapt to visual experience. For example, in rodent primary visual cortex, while individual cells are already orientation-selective at eye opening^21^, their receptive fields remain highly labile over development, especially during the so-called critical period. A striking example is binocular matching, whereby binocular neurons have initially discordant orientation preferences for either eye, but gradually reconcile their binocular orientation preferences over the critical period^28^. In fact, visual selectivity (for example, ocular dominance) retains plasticity throughout adulthood^29^,^30^. Therefore, any model of emerging attractor connectivity in sensory cortex must accommodate the joint emergence and maintenance of precise receptive fields for individual cells. To our knowledge, there is currently no model for the joint development of attractor network connectivity and realistic feedforward receptive fields within a given network.

Here we investigate this problem through computational modelling of developing mouse primary visual cortex. Our main result is that realistic synaptic plasticity, mutual inhibition and exposure to natural stimuli are jointly necessary and sufficient to produce the emergence of competitive neural groups with attractor dynamics, as well as realistic feedforward receptive fields. We built a model of a small patch of cortex, containing 100 principal neurons (similar to the field typically captured by calcium imaging experiments^5^,^7^), with indiscriminate, random connections to and from a local pool of 20 inhibitory neurons, in which both lateral and feedforward excitatory connections are subject to spike-timing-dependent plasticity (Fig. 1). We exposed this model to subregions of natural images pre-processed to emulate retinal filtering. We found that this model spontaneously self-organizes into an attractor network, such that network responses to stimuli tend to fall within a small repertoire of possible multi-cell patterns, reflecting the formation of neuronal groups. Importantly, the learned receptive fields of single cells show the expected ‘oriented-edge' tuning, showing that group behaviour does not sacrifice individual selectivity. Spontaneous (stimulus-absent) activity exhibits the same firing patterns as stimulus-evoked responses (though at a much lower rate), and mixtures of stimuli tend to evoke ‘all-or-none', discrete responses, with sharp transitions, rather than mixed responses, demonstrating the internally generated nature of these patterns. Furthermore, network dynamics remained fast, showing little persistence of activity after stimulus offset. Our model allows us to make predictions about the results of network manipulations which, if confirmed experimentally, might conclusively demonstrate the presence of connectivity-driven attractor dynamics in primary sensory cortex.

## Results

### Emergence of cell clusters

We exposed the network to 1,000,000 image stimuli, with each presentation taking 300 ms of simulated time, separated by 50ms periods of null input. The high number of stimuli was chosen to ensure stabilization (correlation between lateral weight vectors at 500,000 and 1,000,000 presentations: *r*=0.98. Feedforward weight vectors: *r*=0.98). We then froze the connections of the network and recorded the model's response to 1,000 further presentations of (different) image stimuli.

**Figure 2:**
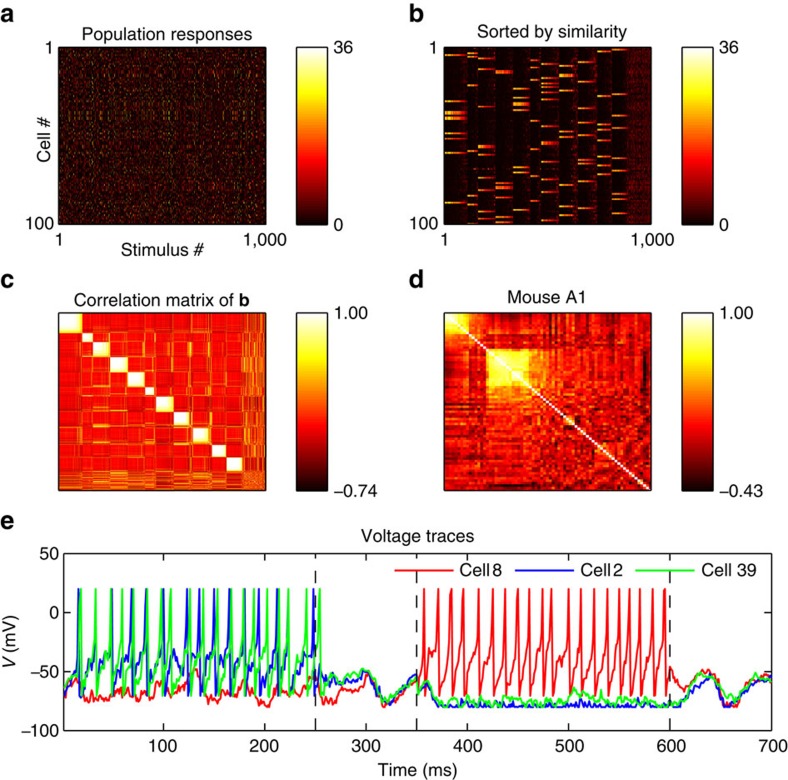
Network responses form discrete clusters as observed *in vivo*. (**a**) Total number of spikes in response to 1,000 stimulus exposures (350 ms duration each), for each cell and each stimulus. (**b**) Same data as in **a**, with columns (network responses) sorted by similarity. The population responses fall within a limited number of discrete patterns. (**c**): Correlation matrix of columns of **b**. Each location *i,j* in this matrix represents the correlation between response vectors *i* and *j* in **b**. Clusters of highly correlated response patterns are readily apparent. (**d**) Correlation matrix of recorded responses in mouse auditory cortex for comparison, redrawn from data provided by Bathellier *et al*.^5^ using the same procedure as **c**. (**e**) Voltage traces of three neurons, two of which (2 and 39) belong to a common cluster and have similar selectivities and receptive fields, while the third (8) has a different selectivity and belongs to a different cluster. The voltage trace covers two stimulus presentation, followed by 100 ms stimulus-absent ‘relaxation' periods indicated by dotted vertical lines (see Methods); the first stimulus is preferred by neurons 2 and 39, while the third is preferred by neuron 8. Neurons 2 and 39 show highly similar, though not identical traces, while cell 8 follows a very different activity pattern.

Figure 2 shows the total responses of all model cells to each successive stimulus presentation, first in their original order (Fig. 2a), then sorted by similarity through hierarchical clustering (Fig. 2b). The correlation matrix in Fig. 2c displays the correlation between the similarity-grouped response patterns in Fig. 2b. The responses show strong clustering, such that the vast majority of population response patterns fall into one of a few different possible response patterns.

**Figure 3:**
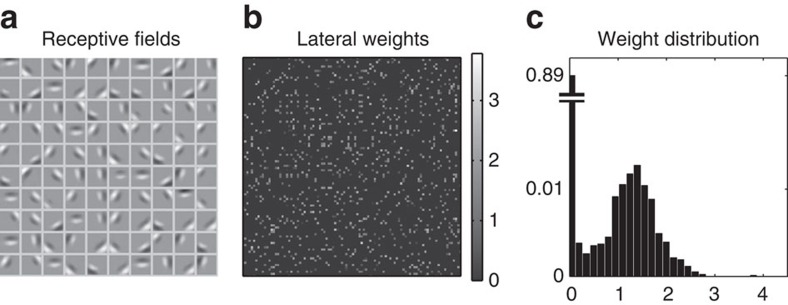
Learned receptive fields and connectivity. (**a**, left) Feedforward receptive fields after development (obtained by subtracting the OFF-centre weight from the ON-centre weight for every input pixel). Following exposure to natural stimuli, the 100 principal cells developed oriented, biphasic receptive fields. (**b**, centre) Connection matrix between the cells (that is, lateral connections). The matrix is sparse and highly symmetrical, revealing strongly bidirectional connectivity. (**c**, right) Distribution of learned E-E weights. Most connections have zero weight, with the non-zero weights forming a single smoothly decaying mode, due to the stabilizing weight-dependent plasticity (see Methods).

Meanwhile, the lateral connectivity is highly bidirectional: the lateral connection matrix correlates highly with its own transpose, excluding the zero diagonal weights (Pearson *r*=0.79, *P*<1e−4) (Fig. 3b). The connectivity is also sparse, with 98% of the total connection weight accounted for by the 10% strongest connections (5% strongest connections: 60% of the sum of all weights). Furthermore, the 5% pairs of cells with the most correlated responses over time represented 50% of the total synaptic connection weight. This is comparable with recently reported results^31^, in which 7% most correlated cell pairs accounted for 50% of the total connection weight. Firing rates for principal neurons show a large range: over 1,000 stimulus presentations, the median principal neuron firing rate was 4 Hz, the absolute maximum firing rate was 122 Hz, and the median maximal response (that is, the median of the set defined by the firing rates of the most active cell for each stimulus) was 58 Hz.

### Individual neurons develop selective receptive fields

The clustering of responses does not compromise the selectivity of individual neurons: the learned receptive fields, reconstructed by subtracting OFF-centre inputs from ON-centre inputs, exhibit the familiar pattern of biphasic oriented-edge detector (Fig. 3a). Mutually connected cells also exhibit highly similar receptive fields: among the 10% most strongly connected pairs (accounting for 98% of the total synaptic weight), the median correlation between both receptive fields of the pair was *r*>0.9. The distribution of learned weights showed a very high peak at zero weight, and a single, smoothly decaying non-zero mode (Fig. 3c), which is qualitatively similar to reports from the literature^32^ (note that, this smoothly decaying mode results from a modification we brought to the voltage-based STDP algorithm—see Methods).

### Evidence of attractor dynamics

**Figure 4:**
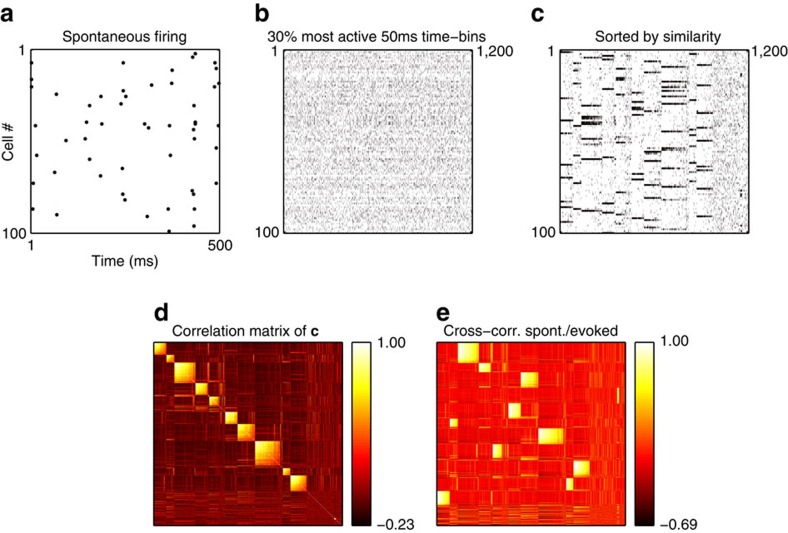
Spontaneous activity patterns recapitulate evoked network responses. (**a**) All spontaneous spikes over 500 ms, showing the sparseness of spontaneous activity. (**b**) Spontaneous spiking activity over 100 s is collected into 50 ms time bins, and the ∼30% most active time bins are selected (almost all time bins have either 0 or 1 spike for each neuron). (**c**) Same data as in **b**, with columns (time bins) sorted by similarity (same procedure as for Fig. 2b). Spiking activity is dominated by discrete patterns, which are the same multi-cell patterns evident in stimulus-evoked network responses, in a different order (Fig. 2b–see also **e**). (**d**) Correlation matrix for the columns in **c**. Clustering of response patterns is as evident as in Fig. 3c. (**e**) Cross-correlation matrix between the columns of **c** and of Fig. 2b. The high correlation between patterns in **c** and Fig. 2b reveals that the firing patterns in spontaneous and evoked activity are highly similar.

Groups of jointly firing neurons do not, by themselves, indicate attractor dynamics, since it is possible that their joint firing might simply be a direct consequence of their similar receptive fields generated by feedforward connections. To demonstrate that the coherent patterns are internally generated, we followed a procedure similar to that used by Miller *et al*.^7^. We recorded spontaneous model activity, in the absence of any stimulus, during a 1,000 ms period; this spontaneous activity is caused by a constant barrage of ‘noise' spikes with a fixed frequency (see Methods). We binned the activity of each cell in 50 ms time bins and then selected the 30% time bins with highest total number of spikes. The resulting correlation matrix of the spontaneous population responses, when sorted by similarity, exhibits the same coherent neural clusters as the stimulus-evoked responses (Fig. 4).

**Figure 5:**
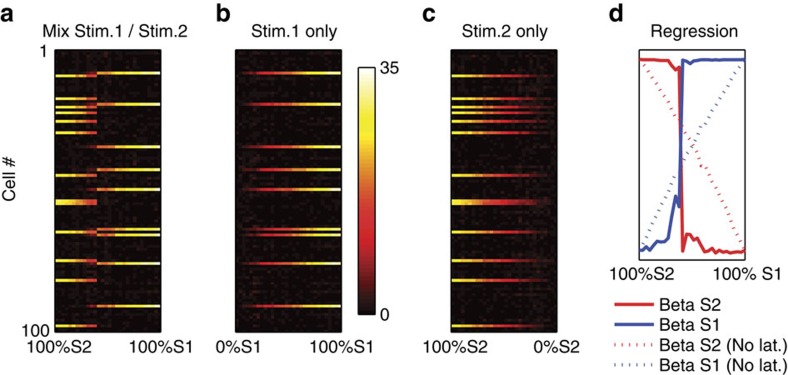
Group responses are discrete and competitive. Model responses to 30 mixtures of two different stimuli (**a**), as well as to identical intensities of either stimulus in isolation (**b**,**c**). Each dot in matrices A–C denotes the total number of spikes of a given neuron for a stimulus presentation. Competitive ‘all-or-nothing' dynamics are evident in the shortness of the ‘mixed response' regime in **a**, as well as the abrupt transition from one response mode to the other (**d**). Disabling all lateral connections results in a smooth transition between response patterns, eliminating the abrupt response switch (**d**, dotted lines).

If the network is driven by attractor dynamics between discrete response patterns, we expect abrupt, nonlinear transitions between response patterns as the stimulus gradually changes from one stimulus to another, as observed *in vivo*^5^. To investigate this question, we followed the same procedure as Bathellier *et al*.^5^. We selected two input patterns, namely those that generated the highest maximum firing across the population in the first and second clusters in Fig. 2c. We then generated 30 linear mixtures of these stimuli, such that the respective proportions of the first and the second source stimulus in the mixture gradually changed from 100% Stimulus 2 and 0% Stimulus 1 to the 100% Stimulus 1 and 0% Stimulus 2. We then recorded the response of the model to each of these mixed stimuli (Fig. 5a). The response to the mixed stimuli shows an abrupt transition between the two response modes evoked by either source stimulus alone. In particular, when the stimuli were combined (Fig. 5a), the range of intensities over which either response pattern occurred was much smaller than for either stimulus in isolation: in Fig. 5b,c (individual, decaying stimuli), the horizontal extent of either response mode is much longer than in Fig. 5a (mixed stimuli). Thus, the abrupt transition is not merely the result of decreasing response to a change in component stimulus intensity, but rather it actually results from competition between the two response patterns. This competitive selection further demonstrates that network responses are driven by internally generated attractor dynamics.

To quantify how much the response patterns to the mixed stimuli resemble responses to either stimulus in isolation, we again used a procedure described by Bathellier *et al*.^5^. We used a simple linear regression model ***r***_*n*_*=β1*_*n*_ ***r***_*1*_*+β2*_*n*_ ***r***_*2*_ , where ***r***_*n*_ is the network response to the *n*th of the 30 mixtures, and ***r***_*1*_ and ***r***_*2*_ are responses to either component stimulus in isolation (after normalizing all vectors to norm 1, to control for differences in overall activity). The *β1*_*n*_ and *β2*_*n*_ series are plotted in Fig. 5d (solid lines), illustrating the sharpness of the transition. Importantly, this abrupt transition was dependent on lateral connections, as the same procedure with disabled lateral connections produced a noticeably shallower transition (Fig. 5d, dotted lines).

**Figure 6:**
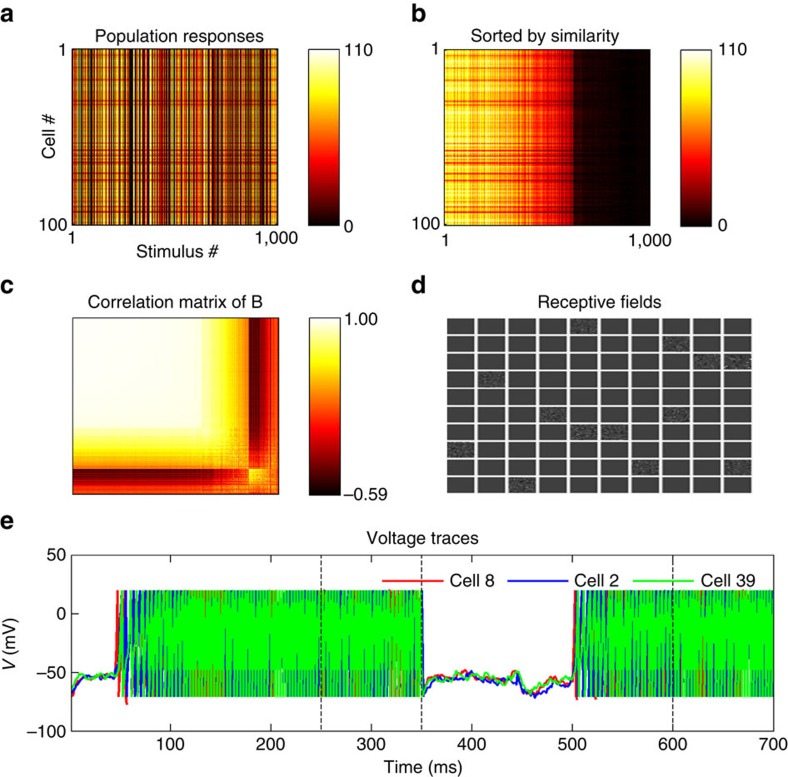
The model does not develop competitive groups or realistic receptive fields when exposed to randomized stimuli. Conventions are as in Fig. 2. Notice the lack of clusters in **c**, the very high firing in **a**, **b** and **e**, and the few non-zero receptive fields (with random, salt-and-pepper structure) in **d**.

To determine the role of stimulus structure in group formation, we ran the exact same model, using the same inputs, but randomly shuffling the pixels in each successive image frame; this preserves the distribution of pixel intensities, while removing spatial correlations present in natural images. When exposed to this randomized input, the model did not develop competitive groups (Fig. 6). Rather, the population simply arranged into a single group of jointly firing cells responding in an all-or-none fashion. Most cells lost all feedforward input, with only a few cells maintaining non-zero receptive fields with random, salt-and-pepper structure. This shows that group formation in the model is dependent on structured stimuli, rather than merely being an automatic by-product of the plasticity algorithm.

### Network mechanisms

**Figure 7:**
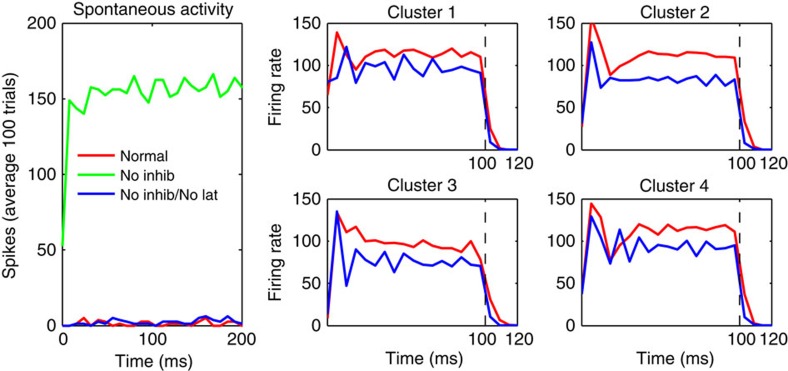
Mechanisms of self-organized network connectivity. (left) Lateral excitatory connections make the network intrinsically unstable in the absence of inhibition. The full network, with lateral connections and mutual inhibition (red curve), produces low spontaneous firing rates. However, when inhibition is removed, but excitatory lateral connections are preserved, the firing rate diverges to a high constant value (green curve). This effect disappears when all lateral connections (both inhibitory and excitatory) are removed, restoring low firing rates (blue curve). Right: average firing rates for a 100 ms stimulus presentation, for each of four cell clusters, using the preferred stimulus for each cluster, both with the full network (red curves) and after removing all lateral connections (blue curves). Dotted vertical lines indicate stimulus offset at *t*=100 ms. Recurrent connectivity results in a small overall amplification, which does not noticeably slow down network dynamics. Notice that all curves quickly decay to zero after stimulus offset.

To investigate which network properties support the model's dynamics, we ran the trained model under altered conditions. First, we disabled all inhibition by silencing all inhibitory neurons, leaving the system otherwise unperturbed. This resulted in very high, self-sustaining firing, even during spontaneous activity (in the absence of any stimulus) (Fig. 7, green curve). This effect disappeared when lateral connections were disabled, demonstrating that the destabilization is caused by the lateral connections (Fig. 7, blue curve). Thus, the excitatory lateral connections make the network unstable in the absence of inhibition. Furthermore, the lateral connectivity did not seem to impose much slowing in the dynamics; on stimulus offset, the cell's activity fell back to zero similarly in the full network and in the same network with disabled recurrent connectivity (Fig. 7, right panels).

### Model predictions

**Figure 8:**
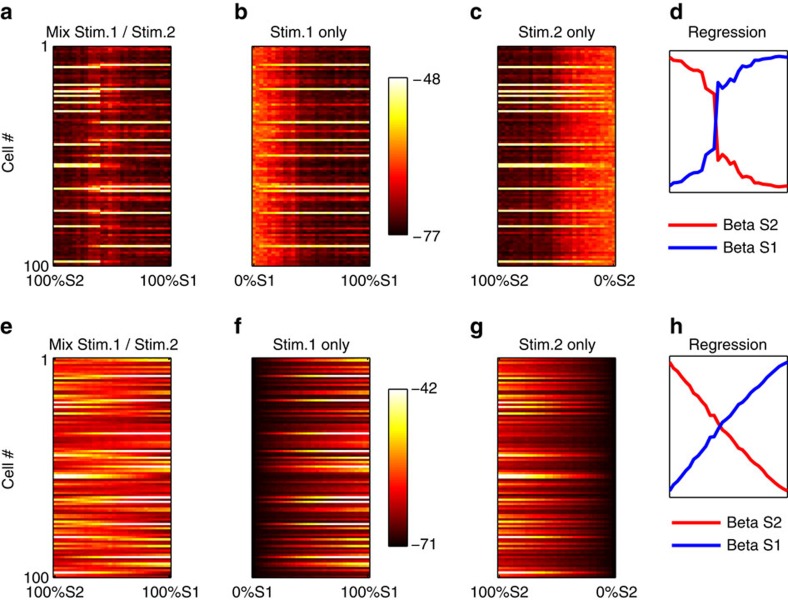
Competitive group dynamics in subthreshold potentials are eliminated by spike blocking. Top panel (**a**–**d**): same information as in Fig. 5, but using the average subthreshold potential of each cell, rather than the total number of spikes, for each presentation. Cell potentials exhibit similar competitive, all-or-nothing behaviour as spikes (Fig. 5) in response to mixed stimuli (**d**). (**e**–**h**, bottom) Same settings, but after blocking spiking activity in all neurons. Competitive dynamics are essentially eliminated, giving way to smooth, gradual transition between response patterns. Thus, competitive group dynamics in the model are due to lateral influences between neurons, which might be tested physiologically.

While these network manipulations confirm the attractor nature of the model network, they might be difficult to translate to physiological experiment. We therefore performed an additional network manipulation, which might be more amenable to physiological implementation *in vivo*, namely, blocking spiking activity^23^,^25^. We first performed the same ‘mixed stimuli' experiment described in Fig. 5, without spike blocking, but using the average subthreshold potential rather than the total number of spikes as the response. We confirmed that the same competitive, all-or-nothing dynamics are also present in subthreshold activity when spikes are allowed in the network (Fig. 8a–d). We then blocked spiking activity in all network cells. This was done by removing the exponential component of the Brette–Gerstner Adaptive Exponential equation (the second summand in equation (1), Methods), which supports the runaway depolarization giving rise to spikes. This manipulation essentially eliminated competitive dynamics in subthreshold potentials, resulting in smooth, gradual transitions from one response patterns to another (Fig. 8e–h). Notice that the subthreshold response patterns to ‘pure', unmixed stimuli S1 and S2 are quite similar with and without spikes (leftmost and rightmost column of Fig. 8a,e); quantitatively, even under spike suppression, both pure stimuli are easily discriminated from each other (Fig. 8h, leftmost versus rightmost regression weights). This shows that spike blocking did not abolish stimulus selectivity in subthreshold potentials, which is largely due to preserved feedforward input. Thus the loss of competitive dynamics is not due to a catastrophic loss of selectivity, but from the loss of mutual influence between cells.

This further demonstrates that the competitive dynamics are due to connectivity between the cells, in a way that might be amenable to physiological experimentation. The model's prediction is that, if competitive group dynamics are indeed the result of cortical attractor dynamics (rather than, say, a mere reflection of upstream processes), then blocking spiking activity should abolish competitive group dynamics in membrane potentials, without eliminating selectivity of individual cells to pure stimuli. Testing this prediction might conclusively determine whether the group dynamics observed in cortical activity^5^,^7^ actually arise from internal cortical dynamics.

### Robustness of results to parameter variation

The model is not critically sensitive to precise parameter values. We ran the model with free parameters increased by 20% (Supplementary Fig. 1; see Methods). The population still organized into competitive clusters, while developing selective receptive fields. Similarly, running the model with parameters reduced by 20% still produced clusters and selective receptive fields, although in this case the receptive fields were much reduced in size. Finally, we also ran an experiment in which we simply quadrupled the number of neurons (400 E, 80 I). The only modification to the model was to divide the common multiplier of all lateral connection weights (*A*_*lat*_—see Methods) by 4, in order to provide a similar regime of overall recurrent inputs relatively to the (unchanged) feedforward inputs. All other parameters were left unchanged. Again, the population arranges into coherent clusters of jointly firing neurons, while still developing realistic receptive fields (Supplementary Fig. 2).

## Discussion

Our results show that a model of a small patch of cortex, endowed with synaptic plasticity (both for feedforward and lateral connections) and all-to-all inhibition and exposed to naturalistic visual stimuli, will spontaneously organize into coherent, mutually competitive groups of jointly firing neurons. Our study provides three main contributions:

1. We demonstrate that realistic plasticity, mutual inhibition and exposure to natural stimuli, are jointly necessary and sufficient to develop fast, non-persistent attractor dynamics in primary sensory cortex, while preserving realistic selectivity of individual neurons. This provides a straightforward explanation for the emergence of ‘cortical ensembles'^7^ or ‘response modes'^5^ observed by *in vivo* imaging of cortical microcircuits.
2. By simulating network manipulations, we make testable predictions which, if confirmed physiologically, could definitely establish whether or not group behaviour in neural responses arises from intra-cortical attractor dynamics.
3. To our knowledge, our model provides the first example of joint development of attractor network connectivity and realistic, orientation-selective feedforward receptive fields in a spiking network simulation.

These neuronal groups^33^ are internally generated by network dynamics. They do not simply result from similar receptive fields caused by mutual influence during development; neither do they merely reflect competitive processes in upstream stages. For example, the same groups observed in stimulus-evoked responses also dominated the spontaneous, stimulus-absent activity, as observed in rodent local circuits^7^, ferret multi-cell activity^10^ and, at larger scales, in cat visual cortex^8^. In addition, mixed stimuli resulted into all-or-none abrupt transitions, even at respective intensities for which either stimulus alone evoked a (different) network response, as observed in mouse auditory cortex^5^. This last behaviour was lost under spike blocking (Fig. 8). This and other network manipulations (Figs 6, 7, 8) support the existence of true attractor dynamics within cortical networks.

An important aspect of our model is the paucity of assumptions. We used state-of-the-art algorithms both for individual neuron dynamics and for synaptic plasticity. Inhibition was modelled as nonspecific, with similar and indiscriminate connections to and from excitatory cells, following current evidence for rodent cortical organization^19^,^20^. Input stimuli were taken from natural images. Our model does not require short-term plasticity, inhibitory plasticity or heterosynaptic scaling, in contrast with related models oriented towards persistent activity and memory function^15^,^17^. The fact that realistic STDP and mutual inhibition suffice to generate attractor networks with fast, competitive group dynamics, suggests that these dynamics might be found in many more cortical sites than those reported so far.

In rodents, primary visual cortex shows significant orientation selectivity at eye opening^21^. However, feedforward receptive fields are highly plastic, as evidenced by binocular matching of orientation selectivities during the critical period^28^ and ocular dominance plasticity in adulthood^29^,^30^. Thus lateral connectivity and feedforward connectivity must adapt to each other in addition to external stimuli. In our model, we decided to simply initialize our feedforward receptive fields with random weights, to avoid additional assumptions about the mechanism of initial orientation selectivity development. This arguably represents the more challenging extremum of potential choices, since any initial selectivity in individual neurons can only facilitate the emergence of appropriate lateral connectivity through Hebbian learning. Thus, we believe that the existence of early orientation selectivity reinforces the conclusions of our study.

By organizing into discrete groups, cortical microcircuits seem to sacrifice much representational power. A population of N independent cells could in principle represent on the order of 2^*N*^ different signals, as opposed to the order of 1–10 reported here and in experimental studies^5^. What could be the compensating advantage of group dynamics? Obvious possibilities include an increase in reliability in the face of massive cortical noise, as well as better control of downstream targets by groups as whole rather than isolated neurons. In addition, we speculate that a major advantage of group dynamics is to vastly accelerate the readout of sensory representation. Measuring the firing rate of a single neuron requires integrating its spikes over a certain period of time, which may impose a large delay on reliable estimation (especially for neurons with low firing rates, as is commonly the case in superficial layers of primary sensory cortex). By contrast, estimating the overall activity of a large population of neurons requires much less time, since one can simply count the number of spiking neurons firing within a short interval (essentially replacing temporal integration with spatial integration). This might explain how large populations of neurons can respond to a change in input current extremely fast, within the first few milliseconds of stimulation—much faster than individual membrane voltage dynamics^34^. Thus, the organization of neural activity into discrete collective patterns might conceivably represent a sacrifice of potential representativity in exchange for much faster decoding by downstream neurons—trading discriminative power for temporal precision.

In conclusion, our results demonstrate that developing cortex can self-organize into attractor networks with fast, competitive dynamics, influencing the selectivity of individual neurons without compromising it, and support the long-standing hypothesis that neurons operate as competitive groups rather than individual analysers. It is interesting to note that this proposal, initially made on purely theoretical grounds^33^, is currently accruing support from multiple streams of evidence.

## Methods

Here we provide a full description of our model. The full software, with source code and detailed instructions, is publicly available at http://github.com/ThomasMiconi/V1stdp.

The model is composed of a single layer of 120 fully connected spiking neurons (100 principal or excitatory neurons, labelled ‘E'; and 20 inhibitory neurons, labelled ‘I'), each of which receives a time-varying input specified by the stimulus. All neurons are simulated as adaptive exponential (AdEx) integrate-and-fire neurons^35^. Mutual connections between principal neurons, as well as feedforward connections from stimulus input to principal neurons, undergo spike-timing-dependent plasticity according to a modified version of the voltage-dependent STDP algorithm from Clopath *et al*.^36^, as described below. By contrast, all inhibitory connections (E-I, I-I and I-E) are non-plastic, with weights independently taken from a uniform distribution at the start of the experiment. Every neuron sends connection to and receives connection from every other neuron. This implies non-selective connections to and from a local pool of interneurons, in accordance with current experimental evidence^19^,^20^. Feedforward connections are initialized to random values, while all lateral E-E connections are initially set to weight zero. In accordance with Dale's law, all synapses from excitatory neurons have positive weights, while all synapses from inhibitory neurons have negative weights.

The stimuli are square image subregions, of size 17 × 17 pixels, extracted at random locations (uniform sampling with replacement) from a set of natural images, and processed with difference-of-gaussian filters (with s.d. 1 and 2 pixels) to emulate centre-surround retinal responses. The patches are randomly rotated to avoid biases in orientation distribution, and individually mean-subtracted and scaled to the [−1,1] intensity range to emulate luminance and contrast adaptation. We then duplicate these patches into two linear vectors of 17 × 17 values each; the first vector has all negative values set to zero, and constitutes the ON-centre inputs to the network. The second vector, which has all positive values set to zero and all remaining values changed to a positive sign, constitutes the OFF-centre inputs to the network.

Thus, each principal neuron receives 17 × 17 × 2 feedforward connections (one for each ON-centre and OFF-centre input), as well as 119 lateral connections (autapses are disallowed).

Our implementation of neural dynamics and plasticity is based on refs 21, 35, 36, with important differences as described below. In the following description we use the nomenclature of refs 35, 36. Each neuron has a membrane potential *u*(*t*) that varies according to the following equations:


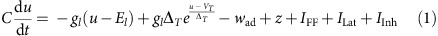



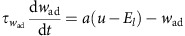



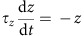



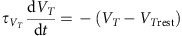


*C* is the membrane capacitance, *g*_*l*_ is leak conductance, *E*_*l*_ is resting potential, Δ_*T*_ is the so-called ‘slope factor' and *V*_*T*_ is an adaptive ‘threshold' (that is, the value of *u* beyond which a spike is irreversibly engaged even in the absence of additional input), which decays exponentially to a resting value *V*_*T*rest_ in the absence of spikes. *Wad* is a hyperpolarizing adaptation current which increases on each spike (see below) and decays exponentially otherwise. *Z* is a spike afterpotential, set to a fixed value *I*_sp_ on each spike (see below) and decaying exponentially. *I*_FF_ is the feedforward input determined by the current stimulus, *I*_Lat_ is the later excitatory input coming from recurrent connections, and *I*_inh_ is the inhibitory input (see below).

When *u(t)* reaches a certain high potential *V*_PEAK_, a spike is deemed to have occurred and the following discrete adjustments take place (in addition, *u* is clipped to *V*_PEAK_ for one timestep, equivalent to 1ms of simulated time; see below):


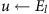



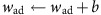



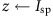



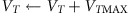


The feedforward input *I*_FF_ is the weighted sum of the incoming feedforward stimulus-evoked spikes. The incoming feedforward spikes are contained in a vector of 17 × 17 × 2 independent Poisson spike trains, whose rates at any time are set by the concatenating the ON-centre and OFF-centre stimulus inputs described above. This vector is multiplied (dot-product) by the cell's vector of 17 × 17 × 2 feedforward weights *w*_FF_, resulting in the total feedforward input for this cell *I*_FF_.

The lateral, recurrent input *I*_Lat_ is computed in a similar way, but uses the vector of outgoing spikes from other cells, both excitatory and inhibitory, received at the current timestep (taking into account the random delays associated with each synapse), multiplied (dot-product) by the vector of lateral weights *w*_Lat_ and multiplied by a constant factor *A*_lat_=5.0. Again, note that there is no self-connection, so cells do not receive excitation from their own spikes.

Inhibition occurs through recurrent connections between excitatory cells (E) and inhibitory cells (I). E and I cells are modelled according to the same equations. While recurrent weights between E cells are plastic, as are feedforward weights from retinal input to E cells (see below), recurrent weights to and from inhibitory cells are taken from a uniform distribution with minimum 0 and maximum *W*_max*I-I*_*, W*_max*I-E*_ or *W*_max*E-I*_ depending on the identity of pre-synaptic and post-synaptic neurons. All weights from inhibitory cells are then switched to negative sign. These inhibitory weights then remain fixed for the duration of the experiment, as opposed to the plastic feedforward and E-E weights (see below).

All delays between cells are taken at the start of the experiment from an exponential distribution with median 4 ms, with a hard minimum of 1 ms, and remain fixed for the whole experiment.

In addition, each cell also receives noise inputs as Poisson trains of excitatory spikes, with mean rate 1,800 Hz, respectively. This allows for non-zero spontaneous activity, as shown in Fig. 4.

Neural plasticity is implemented according to an adapted version of the Clopath–Gerstner voltage-dependent STDP algorithm^36^. Feedforward and lateral E-E weights are modified jointly, without any special distinction between the two (again, all synapses to or from inhibitory cells are non-plastic). Initial feedforward weights are chosen from a uniform random distribution, while initial lateral E-E weights are set to zero. Long-term potentiation (LTP) and long-term depression (LTD) are implemented separately and independently. LTD is governed by the arrival of pre-synaptic spikes according to the following equations:


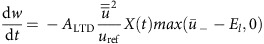



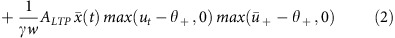



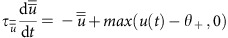



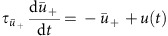



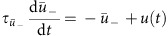



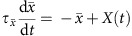


*w* is the weight of a given synapse. *u*_*+*_*, u*_*−*_ and 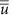 are three different exponential traces of voltage *u(t)*, with respectively a short, slightly longer and much longer time constant; the former two are used in immediate modifications in response to spikes, while the third is used for long-term homoeostatic adaptation, as described below. In addition, note that 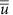 actually registers how much *u(t)* lies above the reference value *θ*_+_ (which is set close to firing threshold), rather than raw voltage *u(t)*.

*A*_LTD_ and *A*_LTP_ are two multiplicative constants. X(t) is a binary value indicating whether or not a pre-synaptic spike arrived at this synapse at time *t*, and 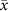 is an exponential trace of this binary variable. *θ*_+_ is a plasticity threshold, set to a constant approximation of the (variable) firing threshold *V*_*T*_, namely (*V*_*T*MAX_+V_*T*rest_ )/2. ‘max(*x*, 0)' denotes half-rectification, equal to *x* if *x*>0 and 0 otherwise. *u*_ref_ and *γ* are two constant scaling factors discussed below.

Intuitively, the equation above can be summarized as follows: LTD occurs if a pre-synaptic spike occurs (*X*(*t*)>0), proportionally to recent depolarization (trace of membrane potential *u*_*−*_ above resting potential), and to the square of long-term above-threshold activity (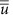). The latter, long-term component has a homoeostatic function by making LTD a superlinear function of long-term activity. LTP occurs if membrane potential exceeds a high threshold close to firing threshold, while the cell was recently depolarized, and a pre-synaptic spike occurred in the recent past (as indicated by 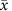); also, LTP is inversely proportional to current weight value *w*, with scaling factor *γ* (see below).

A potential pitfall is that the above equations are sensitive to the random fluctuations in the exponential runaway stroke of the AdEx voltage evolution, which may vary greatly in a fixed-timestep forward-Euler simulation. To alleviate this source of variability, we adapt a method used in original code by Clopath *et al*. (obtained from Claudia Clopath). Every cell that is detected as spiking has its voltage *u* clipped to a fixed value *V*_peak_ for 1 ms, then reset to *E*_*l*_. Thus, the shape of the voltage trace for each spike is highly stereotyped, especially considering that only the portion of the voltage trace above the maximum threshold value (that is, the ‘crest' of each spike) is taken into account for the calculation of potentiation and long-term homoeostatic traces ( equation (2)).

Besides numerical adjustments to a few parameters, there are two main differences with Clopath *et al*.^36^. The first difference is that LTP is weight-dependent: the magnitude of LTP (but not LTD) varies with current weight value, due to the 1*/γw* factor: larger weights are more difficult to increase. This replaces the hard clipping of weights within a fixed range used in the original Clopath–Gerstner algorithm. The main reason for this change, besides increased realism, is that we found the unmodified Clopath–Gerstner algorithm to be strongly ‘saturating' in the face of natural image stimuli: it tends to set all feedforward weights to either the minimum value (that is, 0) or maximum value. This results in unrealistic black-and-white receptive fields with no graded weights, the size of which depends on the allowed maximum weight (setting a high maximum weight leads to small RFs, since fewer inputs are needed to generate the same amount of firing). Note that a similar effect was apparent in Fig. 8 of Clopath *et al*.^36^. With weight-dependent LTP, we can dispense with hard maximal values and obtain more realistic, graded receptive fields, without adding new parameters (the scaling parameter *γ* replaces the discarded hard maximum parameter), as shown in Fig. 3c. Notice that weights are still hard-clipped from below at the zero value.

The other difference is that 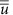 is a long-term exponentially decaying, long-term (20 s) trace of above-threshold activity, rather than a hard average of raw depolarization over 1 s. We found that this change helped stabilize simulations, by making the homoeostatic component more closely related to actual firing activity.

Our numerical parameters were largely taken from existing refs 21, 35, 36 and are described in Supplementary Table 1. We simulated the network's dynamics by simple forward integration of these equations (Euler method) with a timestep of d*t*=1 ms.

For the experiment with quadrupled number of neurons (Supplementary Fig. 2), all parameters were exactly identical, except that the common multiplier of lateral inputs *A*_lat_ was also divided by four to ensure a similar regime of recurrent inputs. For the experiment with varying parameter values (Supplementary Fig. 1), *A*_lat_, *W*_max*I-I*_, *W*_max*I-E*_, *W*_max*E-I*_, *γ*, *A*_LTP_ and *A*_LTD_ were all increased or decreased by 20%.

### Data availability

The full source code for our model, as well as instructions for replication, is available at http://github.com/ThomasMiconi/V1stdp.

## Additional information

**How to cite this article:** Miconi, T. *et al*. Spontaneous emergence of fast attractor dynamics in a model of developing primary visual cortex. *Nat. Commun.* **7,** 13208 doi: 10.1038/ncomms13208 (2016).

## Supplementary Material

Supplementary Information Supplementary Figures 1-2 and Supplementary Table 1

## Figures and Tables

**Figure 1:**
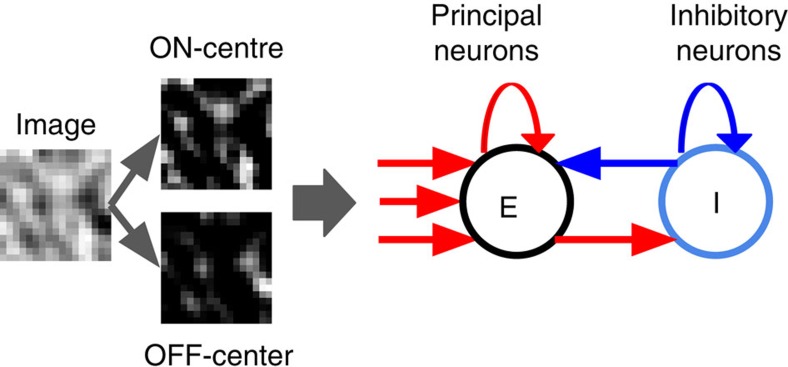
Organization of the model. We model a small local field of rodent V1 cortex, in which all cells have similar retinotopy. Input images (17 × 17 pixels in size) are first processed through an ON-centre and an OFF-centre surround filter, generating a total of 17 × 17 × 2 inputs (each of which emulates an ON-centre or OFF-centre retinal ganglion cell). Each principal neuron (E) receives one excitatory connection from each retinal input, as well as one excitatory connection from every other principal neuron. In addition, principal neurons send excitatory connections to a population of inhibitory interneurons (I). Inhibitory neurons send inhibitory connections to principal neurons and to each other. Feedforward and E-E connections are plastic; E-I, I-I and I-E connections are fixed. See Methods for a full description of the model.
