# Supplementary material for: Spontaneous emergence of fast attractor dynamics in a model of developing primary visual cortex
Source: Nat Commun. 2016 Oct 31;7:13208. doi: 10.1038/ncomms13208 (PMC5095518; doi:10.1038/ncomms13208)
Supplement: Supplementary Information — Supplementary Figures 1-2 and Supplementary Table 1 [file ncomms13208-s1.pdf]

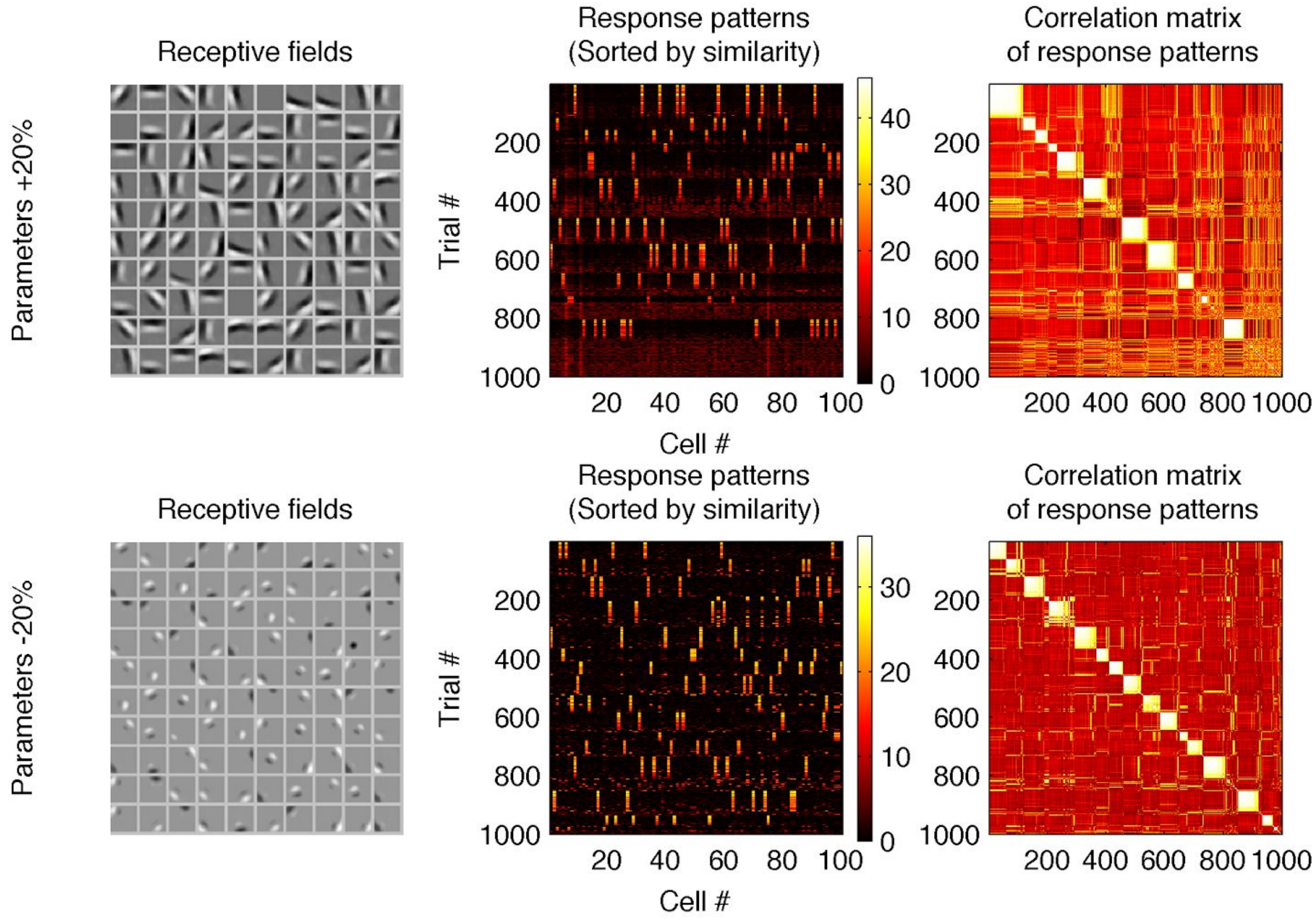

Supplementary Figure 1: Model response to moderate variation in parameters. Top row: after increasing free parameters by 20%, the model's behavior is largely unchanged, except for larger receptive fields. Bottom row: reducing all parameters by 20 % strongly reduces receptive field size, while preserving clustering behavior.

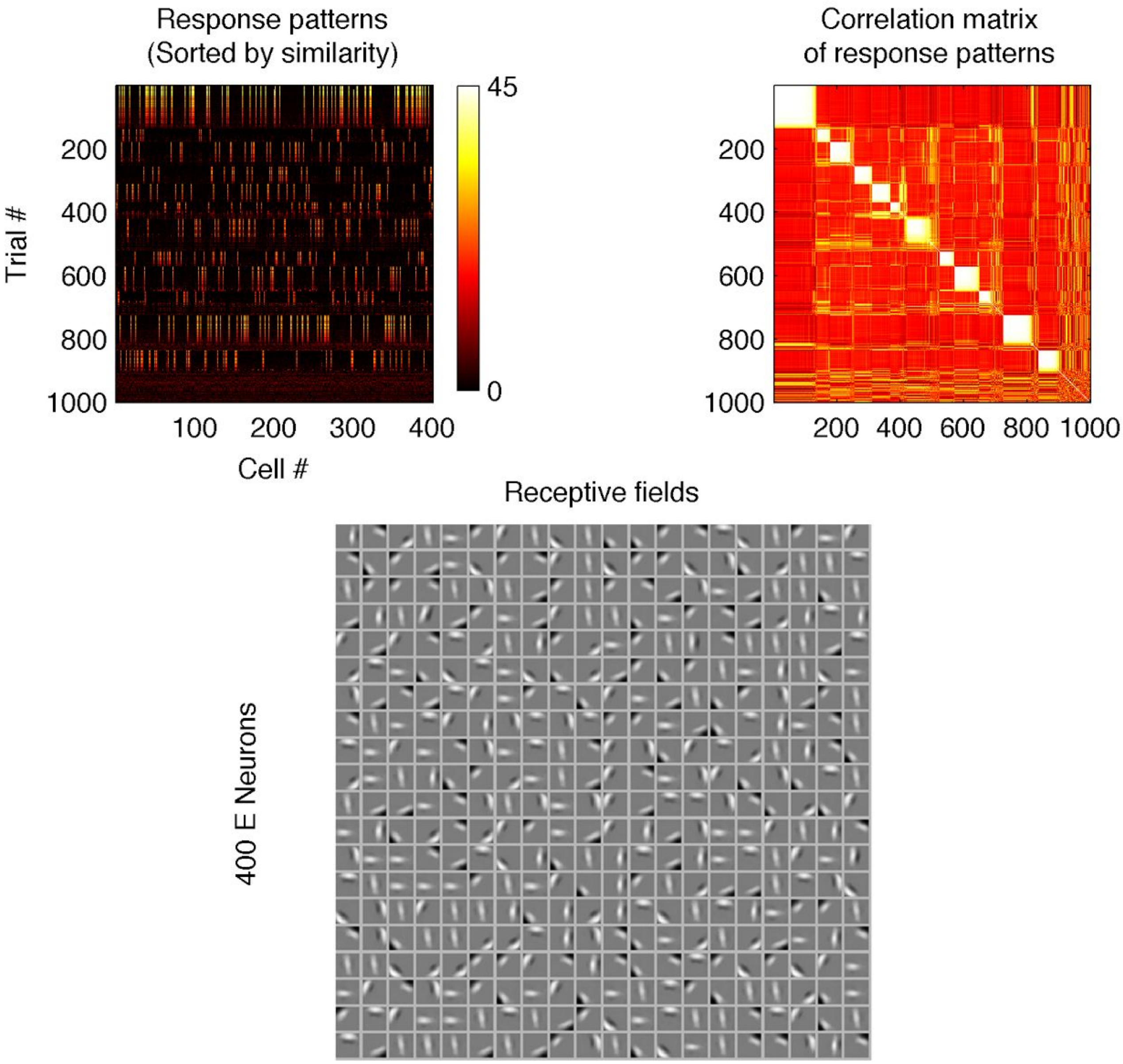

Supplementary Figure 2: Model behavior is robust to network expansion. Quadrupling the number of neurons (400 E, 80 I) does not affect the conclusions of the model.

C	281
g_l	30nS
E_l	-70.6mV
ΔT	2mV
a	4nS
b	0.805pA
V_{Trest}	-50.4mV
V_{Tmax}	-30.4mV
I_{sp}	400pA
τ_z	40ms
τ_{wad}	144ms
τ_{VT}	50ms
τ_{u-}	10ms
τ_{u+}	7ms
τ_x	15ms
W_{EImax}	20
W_{IImax}	0.2
W_{IEmax}	0.2
θ_{u+}	-45.3mV
θ_{u-}	-70.6mV (= E_l)
u^2_{ref}	50
A_{LTD}	$2.1*10^{-4}$
A_{LTP}	$4.8*10^{-7}$
$\tau_{\bar{\bar{u}}}$	20s
A_{lat}	5.0
	0.33

Supplementary Table 1. Model parameter values. The full source code is available at https://github.com/thomasmiconi
